# Association of *Toxoplasma gondii* infection with hematological parameters and CD4^+^ cell counts among pregnant women attending antenatal care in a public hospital of Northwest Ethiopia

**DOI:** 10.1016/j.htct.2026.106480

**Published:** 2026-06-02

**Authors:** Eden Woldegerima, Mastewal Birhan, Mequant Melese, Destaw Fetene Tesshome, Mequanente Dagnaw, Tadelo Wondmagegn, Nega Berhane

**Affiliations:** a Department of Medical Biotechnology, Institutes of Biotechnology, University of Gondar, Gondar, Ethiopia; b College of Veterinary Medicine &Animal Sciences University of Gondar, Gondar, Ethiopia; c Department of Obstetrics and Gynecology, School of Medicine, University of Gondar, Gondar, Ethiopia; d Department of Epidemiology and Biostatistics, Institute of Public Health, University of Gondar, Gondar, Ethiopia; e Department of Immunology & Molecular Biology College of Medicine & Health Sciences University of Gondar, Gondar, Ethiopia

**Keywords:** *Toxoplasma gondii*, Hematological parameters, CD4^+^ cell count, Pregnant women, Ethiopia

## Abstract

**Background:**

*Toxoplasma gondii* affects one-third-of the global population and may alter hematological parameters and CD4^+^ T cell counts, especially during pregnancy. This study evaluated the association of *T. gondii* with alterations in hematological values in pregnant women attending a public antenatal care hospital in Northwest Ethiopia.

**Methods:**

An analytic cross-sectional study of 554 pregnant women (301 seropositive, 253 seronegative) attending antenatal care at a public hospital from 2022 to 2023 assessed T. gondii exposure using ELISA IgG/IgM kits (Human Diagnostics, Germany). Blood samples collected in EDTA tubes were analyzed for hematological profiles using a Coulter Hematology analyzer, and the CD4^+^ cell count with a BD FACSPresto™. Data were analyzed with SPSS 21.0. Descriptive statistics and independent sample t tests were performed: normality was confirmed using the Kolmogorov-Smirnov test

**Results:**

Significant differences were observed in hematological values (white blood cell count, hemoglobin, hematocrit, red blood cell count, lymphocytes, neutrophils, and mean corpuscular volume) between seropositive and seronegative women (p-value < 0.001). Platelet counts showed no significant variation (p-value = 0.811). However, CD4^+^ cell counts were significantly lower in toxoplasmosis-infected women (p-value < 0.001).

**Conclusion:**

*Toxoplasma* gondii infection is associated with alterations in hematological parameters and immunological profiles in pregnant women. Routine screening during antenatal care and preventive education are recommended.

## Introduction

The parasite *Toxoplasma gondii* is a single-celled, eukaryotic, coccidian protozoan that must live inside cells [1]. It is in the group of phylum Apicomplexa and relies on the actomyosin system, an active process that invades host cells. Several protozoan parasites utilize the actomyosin system, causing serious illnesses in a range of hosts, including humans and other warm-blooded domestic and wild animals [2]. *T. gondii* is a prevalent zoonoses, affecting one-third-of the global population [3,4]. It causes toxoplasmosis, a potentially fatal disease in immunocompromised patients, and can lead to congenital defects in the fetuses of women infected during pregnancy [5,6]. Unlike other coccidian, it can infect any nucleated cell, including immature red blood cells (RBC) in warm-blooded animals [7].

*T. gondii* has a complex life cycle, infecting most warm-blooded species. Similar to *Plasmodium*, it infects cells by invading the host cell membrane or disrupting the cytoskeleton, often resulting in cell rupture [8,9]. This process, known as cell lysis, occurs during the parasite's exit [10]; The exact mechanism of the exit is still unclear, but it is a facultative heterogeneous process, involving sexual and asexual phases in different hosts [11,12].

*T. gondii* infection can occur in two stages: acute toxoplasmosis, which is often asymptomatic in healthy adults, and chronic persistence of cysts in immune-competent individuals [13]. Chronic infection and cell-mediated immunodeficiency can cause toxoplasmosis symptoms to reoccur and lead to a decrease in the CD4^+^ cell count [14]. Immunocompromised individuals may experience headaches, confusion, poor coordination, seizures, lung problems, and blurred vision due to retinal inflammation [15]. Latent toxoplasmosis typically presents with mild symptoms but can cause lesions in the heart, skeletal muscle, and central nervous system [16,17]. In humans, the initial *T. gondii* infection typically does not show any noticeable symptoms. However, some individuals may experience ocular toxoplasmosis or cervical lymphadenopathy [18].

During pregnancy, the parasite can cross the placenta and infect the fetus, posing significant risks to both the fetus and the mother. Vertical transmission from an infected mother can lead to miscarriage, congenital infection, severe fetal damage, retinochoroiditis, and even death or infertility in women [4,12,19].

CD4^+^ T cells play a crucial role in controlling both acute and chronic *Toxoplasma* infections. While CD8^+^ T cells are essential for managing chronic infection, their maintenance depends on CD4^+^ T cells. CD8^+^ T cell exhaustion can lead to reactivation of the infection [20,21]. Non-exhausted antigen-specific CD4^+^ T cells can restore function and prevent infection reactivation [22].

In humans, infection with *T. gondii* induces both cellular and humoral immune responses in immunocompetent individuals. The immune system significantly reduces the number of parasites, although the encysted form survives. Acquired immunity is long-lasting and protects against reinfection. Cellular immunity is a critical component of the host's immune response to *Toxoplasma* infection [23].

Global infection rates of *T. gondii* in pregnant women aged from 18 to ​49 range from 2% to 90% [11]. Risk factors and transmission routes significantly impact the health of pregnant women. Specifically, infection rates correlate with environmental exposure to cat populations and are further influenced by hygiene practices, such as hand washing, sanitation, and the consumption of contaminated food or water [5,12,24]. Accurate detection and the use of sensitive diagnostic methods are crucial for effective control and treatment of infections. This study aims to determine the effect of *T. gondii* infections on hematological parameters and on the CD4^+^ cell count among pregnant women attending antenatal care at a public hospital in Northwest Ethiopia. The findings provide baseline information for planning and implementing control and prevention strategies, as well as for advancing the understanding of the epidemiology and management of toxoplasmosis.

## Materials and methods

### Study design and setting

An analytic cross-sectional study was conducted to assess the effect of T. gondii infection on hematological parameters and on the CD4^+^ cell count among pregnant women receiving antenatal care at a public hospital in Northwest Ethiopia. Serological testing employing an enzyme-linked immunosorbent assay (ELISA) was performed between 2022 and 2023. Participants were categorized into two groups: those who tested positive for toxoplasmosis (seropositive) and those who tested negative (seronegative - control group).

### Study participants

The study population included pregnant women aged 18 to 49 who attended antenatal care during the study period and provided consent to participate. However, pregnant women unable to communicate due to medical conditions and those who were human immunodeficiency virus (HIV)-positive were excluded from the study.

### Data collection tools and procedures

Seropositive participants were identified by positive results for parasite-specific IgG and IgM antibodies using ELISA kits (Human Diagnostics, Germany) following the manufacturer’s instructions. Seronegative participants had negative test results.

Differences in mean values of hematological parameters and CD4^+^ cell count were analyzed between seropositive and seronegative groups to understand how *T. gondii* infections affected these variables.

Qualified phlebotomists collected 3 ml of venous blood from each participant using sterile needles and syringes. The samples were immediately transferred into labeled tubes containing ethylenediaminetetraacetic acid (EDTA) to prevent coagulation. These samples were then used for hematological analysis and CD4^+^ T cell counts within one hour of collection. A Coulter Automated Hematology Analyzer was employed for the comprehensive blood counts.

This analyzer provided quantitative data regarding the total number of RBC, white blood cells (WBC), lymphocytes, neutrophils, and platelets. Additionally, the analyzer measured hematocrit (Hct), hemoglobin (Hb), and mean corpuscular volume (MCV).

This study utilized FACSPresto™ (BD Biosciences), a single-platform automated analyzer designed for point-of-care testing. This device provided absolute CD4^+^ T cell counts, CD4^+^ percentages, and Hb levels.

### Data quality control

Laboratory technologists received a one-day training session that focused on the study's purpose and significance, the registration of laboratory test results, and the importance of maintaining the confidentiality of these results. Standard operating procedures (SOP) and manufacturer instructions were strictly followed throughout the procedures, and all reagents were prepared according to the manufacturer’s instructions. The lead investigator maintained the quality of the test result by strictly adhering to the laboratory SOP, from the pre-analytic phase of blood collection to the post-analytical phase of reporting results. The investigator also frequently checked and ensured adherence to these procedures. Training was provided to laboratory technologists and supervisors to reduce variations in data collection (information bias). This training included standardized instructions on using and interpreting each test.

### Data processing and analysis

Data were entered into EpiInfo, version 7.1.5.2, and then exported to the Statistical Package for Social Science (SPSS) version 21. Normality of distribution was evaluated using the Kolmogorov-Smirnov test. Data cleaning was performed to ensure accuracy, consistency, and the handling of missing values. Descriptive statistics, such as mean and percentages, were used to describe the data, which were then presented using text and tables. An independent t-​test was conducted to determine the mean difference between the seropositive and seronegative groups. A p-value <0.05 and a 95% confidence interval (CI) indicated statistically significance.

## Results

### Participants characteristics

A total of 554 pregnant women receiving antenatal care were enrolled, 301 (54.3%) of whom were seropositive for *T. gondii*. The majority were aged 26–30 (42%) and resided in towns (78.9%) (Table 1).

**Table 1:** Characteristics of pregnant women (n = 554) attending antenatal care in a public hospital of Northwest Ethiopia.

Variable	n (%)	Seropositive n (%)	Seronegative n (%)	p-value
**Age**				
18–20	37 (6.7)	25 (67.6)	12 (32.4)	0.021
21–25	125 (22.6)	64 (51.2)	61 (48.8)	
26–30	233 (42.1)	125 (53.6)	108 (46.4)	
31–35	102 (18.4)	53 (52.0)	49 (48.0)	
36–43	57 (10.3)	34 (59.6)	23 (40.4)	
**Residence**				
Urban	437 (78.9)	218 (49.9)	219 (50.1)	0.003
Rural	117 (21.1)	83 (70.9)	34 (29.1)	
**Occupation**				
Student	40 (7.2)	25 (62.5)	15 (37.5)	0.045
Government	177 (31.9)	89 (50.3)	88 (49.7)	
Daily labor	34 (6.1)	19 (55.9)	15 (44.1)	
Farmer	68 (12.3)	49 (72.1)	19 (27.9)	
Merchant	39 (7.0)	20 (51.3)	19 (48.7)	
Other	196 (35.6)	99 (50.5)	97 (49.5)	
**Education level**				
Illiterate	120 (21.7)	80 (66.7)	40 (33.3)	0.008
Elementary	66 (11.9)	39 (59.1)	27 (40.6)	
Secondary	143 (25.8)	66 (46.2)	77 (53.8)	
Higher education	225 (40.6)	116 (51.6)	109 (48.4)	

### Association of *T. gondii* infection with hematological parameters

*T.* gondii has the ability to infect almost every type of nucleated cell, including immature RBC (erythroblasts). The effect of T. gondii infection on different hematological parameters, including WBC, lymphocytes, neutrophils, RBC, Hb, Hct, MCV, and platelets, was examined. Significant differences were found in the mean values of most of these parameters between seropositive and seronegative participants. However, the platelet counts did not show any significant difference (p-value = 0.811) (Table 2).

**Table 2:** Association of *T. gondii* infection with hematological parameters of pregnant women attending antenatal care in a public hospital of Northwest Ethiopia.

Parameter	Seropositive mean (95% CI)	Seronegative mean (95% CI)	P-value
White blood cells (× 10³/µL)	6.2 (6.0–6.4)	7.1 (6.9–7.3)	<0.001
Lymphocyte (× 10³/µL)	2.03 (2.00–2.05)	2.00 (1.98–2.02)	0.059
Neutrophils (× 10³/µL)	2.01 (2.00–2.02)	2.02 (2.00–2.03)	0.038
Red blood cells (× 10^6^/µL)	3.9 (3.8–4.0)	4.4 (4.3–4.5)	<0.001
Hemoglobin (g/dL)	11.2 (11.0–11.4)	12.1 (11.9–12.3)	0.004
Hematocrit (%)	34.1 (33.8–34.4)	36.8 (36.5–37.1)	<0.001
Mean corpuscular volume (fL)	85.2 (83.9–86.5)	87.1 (85.8–88.4)	0.051
Platelet (× 10³/µL)	218 (210–226)	221 (213–229)	0.811

95% CI: 95% confidence interval.

### Association of *T. gondii* infection with the CD4^+^ cell count

On comparing seropositive and seronegative pregnant women, there was a significant difference in the mean CD4^+^ cell counts with the seronegative group having a significantly higher count (Table 3).

**Table 3:** Association of *T. gondii* infection on the CD4^+^ count of pregnant women Public Hospital Northwest Ethiopia.

Parameter	Seropositive Mean (95% CI)	Seronegative Mean (95% CI)	P-value
CD4^+^ cells/µL	1550 (1500–1613)	1996 (1987–2000)	<0.001

95% CI: 95% confidence interval.

## Discussion

This study highlights a significant association between *T.* gondii infection and hematological alterations in pregnant women. Specifically, women who tested seropositive for *T. gondii* exhibited significantly lower levels of WBC, lymphocytes, neutrophils, RBC, Hb, Hct, and CD4^+^ T cell counts compared to their seronegative counterparts. These hematological disturbances may reflect the parasite’s ability to impair hematopoiesis through direct invasion of progenitor cells or immune-mediated mechanisms [25].

The findings of this study align with earlier reports from Tikrit city [25], Saudi Arabia [26], and Iraq [27] that also documented hematological suppression among infected pregnant women. WBCs play an essential role in controlling toxoplasmosis [28], and the parasite’s ability to invade nucleated cells including immature RBC suggests a plausible pathophysiologic mechanism. The clinical implications are concerning, as such alterations could compromise maternal health and fetal outcomes [28]. Neutrophilia was notably higher in seropositive women, indicating an acute inflammatory response potentially induced by the rapid multiplication of tachyzoites [10,26,29]. Neutrophils, guided by chemokine gradients toward the sites of tachyzoite invasion, are among the first responders during infection [26,29].

Interestingly, while some studies from Thailand [30] and Libya [26] report contrasting findings regarding WBC levels, the present results revealed significant leukopenia in infected cases. This suggests regional variability or potential influence from other confounding factors such as nutrition or co-infections. *Toxoplasma* infection can lead to a significant decline in WBC counts in pregnant women. Because these cells are essential for orchestrating both innate and adaptive immune responses, their depletion may compromise the host's ability to control the parasite [25]. Crucially, *T. gondii* infection was also associated with a marked reduction in CD4^+^ T cell counts. CD4^+^ T lymphocytes are pivotal in orchestrating adaptive immunity and ensuring long-term immunological memory [31]. The findings of the current study support existing evidence that lower CD4^+^ counts in seropositive pregnant women may result from suppressed interleukin (IL)-12 and interferon gamma (IFN-γ) production and impaired cytotoxic T cell activity. This immune suppression renders these women more susceptible to opportunistic infections and may contribute to reactivation of latent toxoplasmosis or other latent pathogens. Although most participants were in the age range of 18–49 years, significant immune alterations were consistent across the spectrum. The observed decline in CD4^+^ T cell levels corroborates previous work [32] and underscores the pathogen’s capacity for immune modulation. Notably, some discrepancies were observed when compared to published studies [33, 34, 35]. These inconsistencies may be attributed to differences in study design, population demographics, or diagnostic methodologies.

Despite its strengths, this study is constrained by its cross-sectional nature, limiting causal inferences. Moreover, potential confounders like nutritional deficiencies or unrecognized infections were not exhaustively assessed. Nonetheless, the consistent pattern of hematological and immunological disruptions in *T. gondii*-positive pregnant women suggests a need for routine screening and timely intervention, particularly in endemic settings. By characterizing the hematological impact and immunologic suppression associated with toxoplasmosis, this research reinforces the importance of early diagnosis and immune monitoring in pregnant women. Proactive management can not only mitigate maternal complications but also reduce the risk of vertical transmission to the fetus.

## Conclusion and recommendation

The study demonstrates that *T. gondii* infections during pregnancy are associated with significant alterations in immune and hematological profiles. We recommend routine screening during antenatal care visits and conducting further research into the implications in humans. Additionally, it is advisable to provide education on prevention and risk factors.

## Funding

This research did not receive any grants from any funding agencies.

## Availability of data and materials

For the sake of maintaining patient confidentiality, the raw data will not be shared. Data supporting this research article are available from the corresponding author or first author on reasonable request.

## Ethics, approval, and consent to participate

Ethical clearance was obtained from the Research and Ethical Review Committee of the University of Gondar with a reference number of VP/RTT/05/280/2022. Written consent was secured from all participants. All methods were carried out in accordance with relevant guidelines and regulations.

## Authors’ contributions

Conceptualization: Eden Woldegerima, Mastewal Birhan, Mequant Melese,Tadelo Wondmagegn, Nega Berhane; Data analysis: Eden Woldegerima, Destaw Fetene Tesshome, Mequanente Dagnaw; Investigation: Eden Woldegerima,Tadelo Wondmagegn, Nega Berhane; Methodology: Eden Woldegerima, Destaw Fetene Tesshome, Mequanente Dagnaw; Project administration: Eden Woldegerima, Nega Berhane; Supervision: Eden Woldegerima, Nega Berhane; Writing - original draft: Eden Woldegerima, Mastewal Birhan, Mequant Melese,Tadelo Wondmagegn, Nega Berhane; Writing - review & editing: Eden Woldegerima, Destaw Fetene Tesshome, Mequanente Dagnaw, Nega Berhane.

## Conflicts of interest

The authors declare that they have no competing interests.
